# Identification of *Escherichia coli* multidrug resistance transporters involved in anthocyanin biosynthesis

**DOI:** 10.3389/fmicb.2024.1357794

**Published:** 2024-04-05

**Authors:** Xia Wu, Rongxia Chen, Ping Liang, Jian Zha

**Affiliations:** ^1^School of Food Science and Engineering, Shaanxi University of Science and Technology, Xi’an, China; ^2^Xi’an Key Laboratory of Antiviral and Antimicrobial-Resistant Bacteria Therapeutics Research, Xi’an, China

**Keywords:** cyanidin 3-*O*-glucoside, efflux transporter, anthocyanin, *E. coli*, multidrug resistance transporters

## Abstract

The anthocyanin compound cyanidin 3-*O*-glucoside (C3G) is a natural pigment widely used in food and nutraceutical industries. Its microbial synthesis by *E. coli* is a promising alternative to the traditional extraction methods. However, part of the synthesized C3G accumulates in the cytoplasm, thus potentially causing growth inhibition and product degradation. Therefore, it is necessary to enhance C3G secretion via exploration of native transporters facilitating C3G export. In this study, we report the screening and verification of native multidrug resistance transporters from 40 candidates in *E. coli* that can improve the extracellular C3G production when using catechin as the substrate. Overexpression of single transporter genes including *fsr*, *yebQ*, *ynfM*, *mdlAB*, and *emrKY* were found to increase C3G production by 0.5- to 4.8-fold. Genetic studies indicated that *mdlAB* and *emrKY* are vital transporters in the secretion of C3G. Our study reveals a set of new multidrug resistance transporters for the improvement of microbial biosynthesis of C3G and other anthocyanins.

## Introduction

Anthocyanins are a group of colorful flavonoids with wide applications in the processing and production of food, cosmetics, and nutraceuticals (Blesso, 2019; Alappat and Alappat, 2020). The industrial production of anthocyanins mainly relies on plant extraction, which is constantly influenced by land supply and climate fluctuations (Rodríguez-Mena et al., 2023). A promising alternative is biosynthesis using engineered microbes as cell factories (Pandey et al., 2016; Yang et al., 2020; Courdavault et al., 2021; Yang et al., 2022).

**Figure 1:**
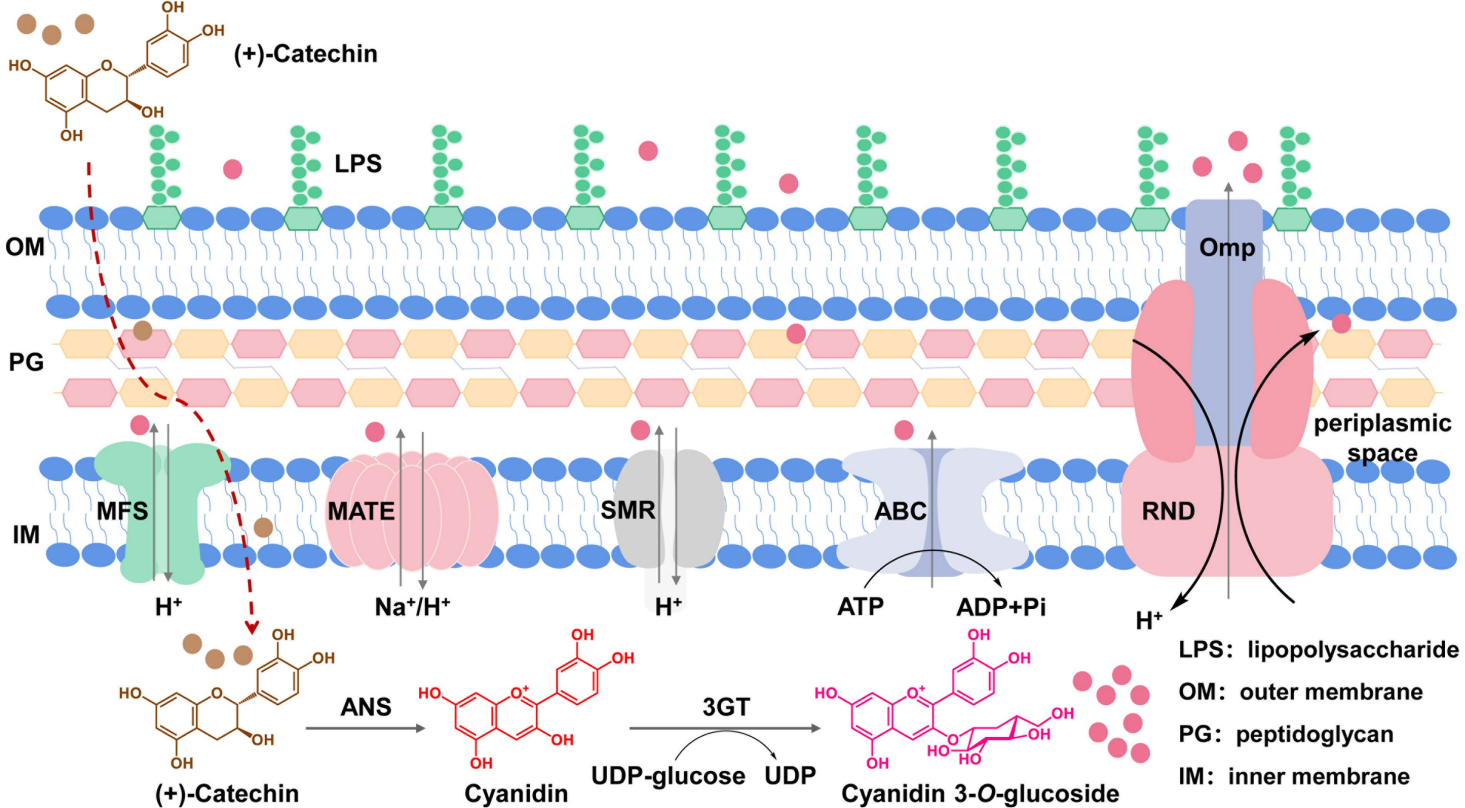
Efflux transporters in *E. coli* cells responsible for transportation of C3G out of cells. C3G is produced by ANS from *Petunia hybrid* and 3GT from *Arabidopsis thaliana* using catechin as the substrate. The produced C3G can be transported out of cells through native MDR transporters located in membranes.

Among all the known anthocyanin compounds, cyanidin 3-*O*-glucoside (C3G) with the highest natural abundance has been a research focus (Belwal et al., 2020). At present, C3G biosynthesis has been achieved in *E. coli*, *Saccharomyces cerevisiae*, *Corynebacterium glutamicum*, and *Lactobacillus* species (Yan et al., 2008; Levisson et al., 2018; Zha et al., 2018; Solopova et al., 2019), with the highest titer (439 mg/L) reported for *E. coli* using (+)-catechin as a precursor (Shrestha et al., 2019). This pathway contains two reactions sequentially catalyzed by anthocyanidin synthase (ANS) and 3-*O*-glycosyltransferase (3GT) using UDP-glucose as a sugar donor (Figure 1). The biosynthesis of C3G in *E. coli* has been engineered on the genetic, enzymatic, metabolic, cellular and fermentation levels for production enhancement (Yan et al., 2008; Lim et al., 2015; Zha and Koffas, 2017; Levisson et al., 2018; Solopova et al., 2019; Zha et al., 2020; Xu et al., 2022). However, a considerable amount of C3G accumulates inside cells possibly due to its high solubility in the aqueous phase and poor capability of diffusing across the cytoplasmic membrane and the outer membrane of *E. coli* (Lim et al., 2015; Zhao, 2015; Behrens et al., 2019). This negatively affects cell growth and metabolism, destabilizes C3G, and complicates the purification process, thereby decreasing the overall C3G titers.

A common approach of tackling the accumulation issue targets transporters to enhance product secretion (Wu et al., 2022). In the long history of evolution aiming at better adaptation to various harsh conditions, microorganisms have developed a set of membrane-bound multidrug resistance (MDR) transporters to pump out harmful substances from the cytoplasm and to reduce their damage to cell components (Jones et al., 2015; Lv et al., 2016). MDR transporters can be classified into five main categories, i.e., the ATP-binding cassette (ABC) superfamily transporters, the major facilitator superfamily (MFS) transporters, the small multidrug resistance (SMR) family transporters, the multi-drug and toxin compound extrusion (MATE) family transporters, and the resistance-nodulation-cell division (RND) superfamily transporters (Figure 1). MDR transporters can export antibiotics, toxic chemicals as well as some natural products (Du et al., 2018). Therefore, engineering of MDR transporters has been adopted in the microbial synthesis of natural products such as amorphadiene, resveratrol, caffeic acid, and reticuline (Zhang et al., 2016, 2017; Hu et al., 2018; Zhao et al., 2018; Yamada et al., 2022; Wang et al., 2023).

In *E. coli*, there is only one native transporter reported to date with C3G-exporting capability, i.e., the ABC transporter YadH, whose overexpression could increase C3G production by 15% (Lim et al., 2015). It is necessary to identify more effective transporters to further improve the bio-production. In our previous study, an MFS efflux transporter named MdtH was identified as a potent anthocyanin transporter, whose overexpression could increase the extracellular C3G level by 110% (Wu et al., 2024), demonstrating the potential of intrinsic *E. coli* transporters in the secretion of C3G. In this study, we constructed a library of all the MDR transporters of *E. coli* and tested their roles in the secretion and production of C3G. We identified a series of potential transporters responsible for C3G production via various mechanisms. Our study provides new targets for the engineering of C3G-producing microbes and will benefit the microbial biosynthesis of related anthocyanins.

## Materials and methods

### Bacterial strains and media

The strains used in this study are shown in Table 1. *E. coli* DH5α was used to construct plasmids and was grown in LB (lysogeny broth) medium supplemented with antibiotics when necessary. *E. coli* BL21 (DE3) was used for C3G production and transporter expression. The plasmid pETM6-At3GT-m-PhANS was transformed into *E. coli* BL21 (DE3) to construct the baseline C3G-producing strain (Cress et al., 2017). The plasmid pACYCDuet-1 was used to express transporter genes. C3G production was performed in M9 medium (pH 5.0) containing 4 g/L of glucose (Lim et al., 2015).

**Table 1:** List of strains and plasmids used in this study.

	Relevant characteristics	Source
**Plasmid**		
pETM6-At3GT-m-PhANS	pETM6 carrying genes *At3GT* and *PhANS*	Cress et al. (2017)
pACYCDuet-1	Double T7 promoter, p15A ori, Cam^R^	Lab stock
pACYCDuet-1-X	pACYCDuet-1 carrying transporter gene X	This study
pCas	Expresses λ Red recombinase Exo, Bet and Gam, temperature sensitive, Kan^R^	Lab stock
pTargetF	J23119 promoter, pBR322 ori, Spc^R^	Lab stock
**Strain**		
DH5α	Cloning of plasmids	Lab stock
BL21 (DE3)	Expression of plasmids and anthocyanin production	Lab stock
Δ*yeb*QΔ*mdlAB*	BL21 (DE3) strain with *yebQ* and *mdlAB* knocked out	This study
Δ*emrKY*Δ*yebQ*	BL21 (DE3) strain with *yebQ* and *emrKY* knocked out	This study
Δ*mdlAB*Δ*emrKY*	BL21 (DE3) strain with *mdlAB* and *emrKY* knocked out	This study

### Construction of plasmids and recombinant strains

The plasmids and primers used in this study are shown in Table 1 and Supplementary Table S1, respectively. The transporter genes were amplified using the genomic DNA of *E. coli* BL21 (DE3) as the template, and subsequently cloned into the plasmid pACYCDuet-1 by *Nde* I and *Xho* I. For the co-overexpression of two transporter genes, the single overexpression plasmids were used and the second gene was cloned into the second ORF between *BamH* I and *Not* I.

The transporter knockout strains were constructed using the CRISPR/Cas9 tool developed in a previous study (Jiang et al., 2015). The knockout strains were formed by homologous recombination between the genome and the homologous arms, with ~200 bp gene sequences deleted in the ORF of the target transporter genes in the repair plasmids. The deletion was finally confirmed by PCR amplification of the extracted genomic DNA from the knockout strains and by gene sequencing of the amplified PCR products. The complementation strains were constructed by introducing the pACYCDuet-1 plasmids carrying the transporter genes into the knockout strains.

For all the cloning work, the enzymes were purchased from New England Biolabs, and kits for plasmid miniprep and gene cleanup were from TIANGEN Biotech. Primer synthesis and gene sequencing were performed by Sangon Biotech.

The plasmid pETM6-At3GT-m-PhANS and pACYCDuet-1 plasmids expressing MDR transporters were co-transformed into *E. coli* BL21 (DE3) using the traditional calcium chloride method.

### Screening of MDR transporters to improve C3G production in 48-well plates

Cells were grown overnight at 37°C in 1 mL LB medium supplemented with 80 μg/mL ampicillin and 25 μg/mL chloramphenicol in 48-deep-well plates (VWR). Overnight cultures (20 μL) were sub-cultured into 1 mL of fresh LB medium containing the necessary antibiotics in 48-deep-well plates. Cells were grown at 37°C with shaking at 220 rpm, induced with 0.5 mM IPTG when OD_600_ (the optical density at 600 nm) reached 0.6, and further grown at 30°C with shaking for 3 h. Cells were then collected by centrifugation at 4,000 rpm and 4°C and resuspended in 1 mL of M9 medium (pH 5.0) with 500 mg/L (+)-catechin. The conversion process was then carried out at 30°C with shaking for 19 h. At the end of the fermentation, 200 μL of the culture supernatants were mixed with an equal volume of acidified methanol and centrifuged at 12,000 rpm for 10 min. The supernatants were used to quantify residual catechin and extracellular C3G.

### C3G production in shake flasks

Glycerol stocks were inoculated into 3 mL of LB medium supplemented with 80 μg/mL ampicillin and 25 μg/mL chloramphenicol and grown overnight at 37°C. The overnight culture (1 mL) was sub-cultured into 50 mL of fresh LB medium containing the necessary antibiotics in 250 mL shake flasks. Cells were grown at 37°C with shaking at 200 rpm, induced with 0.5 mM IPTG when OD_600_ reached 0.6, and further grown for 3 h at 30°C and 200 rpm. Cells were then harvested by centrifugation at 4,000 rpm and 4°C and resuspended in 10 mL of M9 medium (pH 5.0) with 500 mg/L (+)-catechin in 100 mL shake flasks. Cells were incubated at 30°C and 200 rpm for 19 h, and then 200 μL of the culture supernatant were mixed thoroughly with an equal volume of acidified methanol and centrifuged at 12,000 rpm for 10 min. The supernatant was used for the quantification of extracellular C3G and residual catechin.

### HPLC analysis of C3G and catechin

The fermentation products and residual catechin were analyzed by a Shimadzu Essentia LC-16 HPLC system with a diode array detector. A reversed phase C18 column (5 μm, 4.6 × 250 mm) was used to separate and quantify the compounds. The column temperature was maintained at 25°C. Water with 0.1% formic acid (solvent A) and acetonitrile with 0.1% formic acid (solvent B) were prepared as mobile phases at a flow rate of 1 mL/min. The elution program was as follows: 10–40% B at 0–10 min and 40–60% B at 10–15 min. C3G and catechin were detected and quantified by monitoring the absorbance at 520 nm and 280 nm, respectively. The C3G and (+)-catechin standards used in this study were purchased from Sigma-Aldrich.

### Measurement of cell growth of the recombinant strains

Overnight cultures (1 mL) of diverse recombinant strains were sub-cultured into 50 mL of fresh LB medium containing the necessary antibiotics in 250 mL shake flasks, and were grown at 37°C and 200 rpm. Cell growth was monitored periodically by measuring OD_600_ in the absence of IPTG. To study cell growth under the induction condition, cells were grown to OD_600_ of ~0.6 and supplemented with 0.5 mM IPTG. The OD_600_ was then tracked periodically.

## Results

### Screening of potential MDR transporters related to C3G production in *Escherichia coli*

MDR transporters facilitate the outward flow of toxic compounds from the cytoplasm and enhance cell survival under harsh environmental conditions such as exposure to antibiotics and heavy metals (Du et al., 2018). In our previous study, we found that overexpression of an MFS transporter MdtH could increase the extracellular C3G production by around 110% due to better efflux (Wu et al., 2024). To identify other MDR transporters beneficial for extracellular C3G biosynthesis in *E. coli*, we constructed a transporter library containing the known or putative MDR transporter genes (Nishino and Yamaguchi, 2001) in *E. coli* as well as the genes encoding the TolC porin and TolC-involving tripartite efflux systems that are important for drug resistance (Table 2). Some of the MDR transporters that were already analyzed in our previous study were not included here. We overexpressed each of these genes individually under the C3G-producing background and analyzed the extent of extracellular specific C3G production in 48-deep-well plates. Genes that are present in the same operon were overexpressed together. Since the precursor catechin and the product C3G both present antimicrobial activities (Nakayama et al., 2013; Lim et al., 2015) and since the product C3G is unstable under neutral or basic conditions, we adopted a two-step biosynthetic procedure, in which cells were first grown in LB medium and induced for pathway enzyme expression for 3 h, and then shifted to M9 medium (pH 5) containing 500 mg/L of catechin for C3G synthesis.

**Table 2:** Fold increase^a^ in extracellular C3G production per cell OD_600_ in recombinant strains overexpressing diverse groups of MDR transporters in 48-deep-well plates as compared to the control strain.

Transporter family	Overexpressed genes	Fold change of C3G level	Transporter family	Overexpressed genes	Fold change of C3G level
MFS	*emrD*	−0.57 ± 0.08	ABC	*mdlAB*	**1.24 ± 0.24**
	*yajR*	0.12 ± 0.11		*yddA*	**0.40 ± 0.15**
	*fsr*	**1.29 ± 0.70**		*yojIH*	−0.07 ± 0.04
	*mdfA*	−0.71 ± 0.02		*yhiHJ*	**0.57 ± 0.22**
	*mdtG*	−0.86 ± 0.06		*macAB*	−0.21 ± 0.13
	*ydeA*	−0.87 ± 0.02	RND	*acrD*	**0.58 ± 0.06**
	*ydeE*	**1.72 ± 0.14**		*cusCFBA*	**0.37 ± 0.11**
	*ynfM*	**0.57 ± 0.27**		*mdtABCD*	−0.46 ± 0.16
	*ydhC*	**1.60 ± 0.28**		*acrAB*	−0.80 ± 0.13
	*ydiM*	**0.46 ± 0.11**		*acrEF*	−0.99 ± 0.00
	*emrKY*	**0.41 ± 0.14**		*mdtEF*	−0.06 ± 0.02
	*emrAB*	−0.58 ± 0.18	Porin	*tolC*	0.17 ± 0.24
	*mdtNOP*	**0.85 ± 0.03**	TolC complex	*tolC-acrAB*	−0.90 ± 0.03
	*yebQ*	**1.29 ± 0.12**		*tolC-acrEF*	−0.97 ± 0.01
	*bcr*	−0.94 ± 0.03		*tolC-macAB*	−0.94 ± 0.13
	*hsrA*	−0.40 ± 0.07		*tolC-emrKY*	−0.58 ± 0.16
	*Mdtl*	−0.12 ± 0.26		*tolC-emrAB*	−0.91 ± 0.07
	*mdtM*	0.02 ± 0.01		*tolC-mdtABCD*	−0.78 ± 0.01
MATE	*mdtK*	−0.65 ± 0.10		*tolC-mdtEF*	−0.75 ± 0.02
SMR	*sugE*	**0.46 ± 0.14**			
	*mdtJI*	0.05 ± 0.28			
	*emrE*	**1.17 ± 0.63**			

a Fold increase is defined as (C3G level in the overexpression strain—C3G level in the control strain)/C3G level in the control strain. A negative value indicates a decrease in C3G production after the overexpression of the transporter gene. The bold numbers indicate obviously higher extracellular C3G production upon overexpression of the corresponding transporter gene.

As shown in Table 2, overexpression of *fsr*, *ydeE*, *ynfM*, *ydhC*, *ydiM*, *emrKY*, *mdtNOP*, *yebQ*, *sugE*, *emrE*, *mdlAB*, *yddA*, *yhiHJ*, *acrD*, or *cusCFBA* increased the extracellular level of C3G per OD_600_ by 0.37- to 1.72-fold, which was consistent with our hypothesis that the titer of C3G could be improved via efflux enhancement. Therefore, these transporter genes were selected for the subsequent analysis. It is interesting to note that overexpression of the transporter components that form tripartite efflux systems with TolC significantly impeded C3G biosynthesis, and the co-overexpression of *tolC* further enhanced such inhibitory effect, although a higher expression level of *tolC* alone had marginal effect on extracellular C3G concentration.

### Overexpression of *fsr*, *yebQ*, *ynfM*, *mdlAB*, or *emrKY* could significantly increase extracellular C3G production

To verify the role of the selected MDR transporters in C3G biosynthesis, we conducted the bioconversion using the two-stage procedure in shake flasks. It is generally believed that the cultivation conditions in shake flasks can be controlled more stably than those in 48-well plates. Among the 15 selected transporter genes, overexpression of *ynfM* improved the extracellular synthesis per OD_600_ by 4.80-fold compared to the control strain that harbored the empty plasmid (Table 3), and upregulation of *fsr*, *yebQ*, *emrKY* or *mdlAB* increased specific C3G production by 0.54- to 4.12-fold. In comparison, the other transporter genes that were tested effective in 48-well-plates did not show positive correlations at a higher expression level with extracellular C3G production in shake flasks. Such a discrepancy may be attributed to the different extents of oxygen supply and shear stress.

**Table 3:** The fold increase in extracellular C3G production per cell OD_600_ in recombinant strains individually overexpressing selected transporter genes in shake flasks relative to the control strains.

Transporter family	Transporter gene	Fold increase in C3G level
MFS	***fsr***	**1.07 ± 0.13**
	*ydeE*	0.40 ± 0.05
	***ynfM***	**4.80 ± 0.02**
	*ydhC*	0.05 ± 0.20
	*ydiM*	0.04 ± 0.05
	***yebQ***	**4.12 ± 0.48**
	***emrKY***	**0.54 ± 0.01**
	*mdtNOP*	0.26 ± 0.11
	*mdtH*	1.15 ± 0.25
ABC	*yddA*	−0.11 ± 0.11
	*yhiHJ*	0.16 ± 0.02
	***mdlAB***	**2.43 ± 0.10**
RND	*cusCFBA*	−0.25 ± 0.02
	*acrD*	0.38 ± 0.11
SMR	*emrE*	0.19 ± 0.09
	*sugE*	0.15 ± 0.06

Data represents mean **±** standard deviation of three biological replicates. The bold numbers indicate obviously higher extracellular C3G production upon overexpression of the corresponding transporter gene.

### Genes *yebQ*, *mdlAB*, and *emrKY* were strongly correlated with C3G production

**Figure 2:**
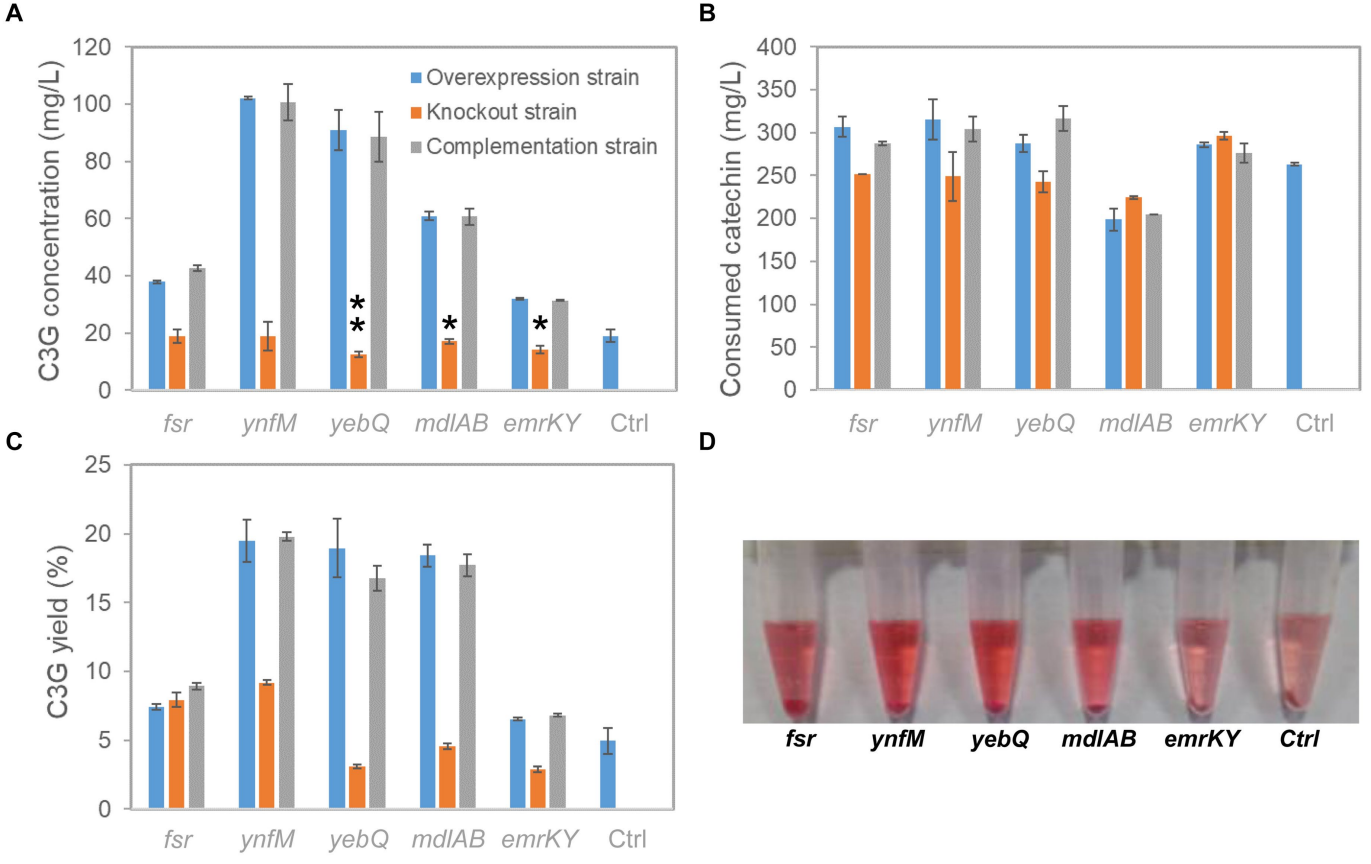
C3G production from catechin by recombinant *E. coli* strains expressing *fsr*, *ynfM*, *yebQ*, *emrKY*, and *mdlAB*. **(A)** The comparison of extracellular C3G titers by the overexpression strains, knockout strains and complementation strains related to transporter genes *fsr*, *ynfM*, *yebQ*, *emrKY*, and *mdlAB*. Statistical analysis was performed using two-tailed student *t*-test. *p < 0.5; **p < 0.01. **(B)** Utilization of catechin by different strains. **(C)** The extracellular yields of C3G from catechin by various strains. **(D)** The picture showing the supernatant of the final fermentation products by the recombinant strains overexpressing *fsr*, *ynfM*, *yebQ*, *emrKY*, and *mdlAB.* Data represents mean ± standard deviation of three biological replicates.

To investigate whether the phenotype of extracellular C3G production was indeed associated with the expression levels of the selected transporter genes, i.e., *fsr*, *ynfM*, *yebQ*, *mdlAB*, and *emrKY*, we constructed single-gene deletion and complementation strains, and analyzed C3G biosynthesis and catechin utilization by various recombinant strains. As shown in Figure 2A, overexpression of these genes greatly promoted extracellular C3G concentration compared with the control strain carrying the empty plasmid; deletion of *yebQ*, *mdlAB*, or *emrKY* decreased C3G production by 10–30% compared with the control strain, whereas deletion of *fsr* or *ynfM* had no impact on extracellular C3G concentration. Complementation of the corresponding genes in the knockout strains restored C3G production to levels similar to those in the overexpression strains. Moreover, despite large variations in C3G production by various overexpression strains, they consumed similar amounts of catechin except the *mdlAB*-related strains that utilized noticeably less catechin (Figure 2B). Taken into consideration both substrate usage and product formation, we found similar extracellular C3G yields obtained by the overexpression of *ynfM*, *yebQ* and *mdlAB*, which were 2.7- to 2.9-fold higher than that of the control strain. Interestingly, deletion of *fsr* or *ynfM* resulted in 0.61- or 0.82-fold higher yields, respectively (Figure 2C).

As C3G is naturally pink under acidic conditions, it is easy to judge the production using the colorimetric assay and by visual observation. Notably, the culture overexpressing *ynfM* or *yebQ* showed darker colors than the control strain and the other overexpression strains (Figure 2D). Additionally, the *yebQ*-overexpressing culture turned pink as early as 1 h after catechin supplementation (Supplementary Figure S1). In comparison, the control culture did not develop any pink color even after 4 h of biosynthesis.

### Effect of transporter overexpression on cell growth

**Figure 3:**
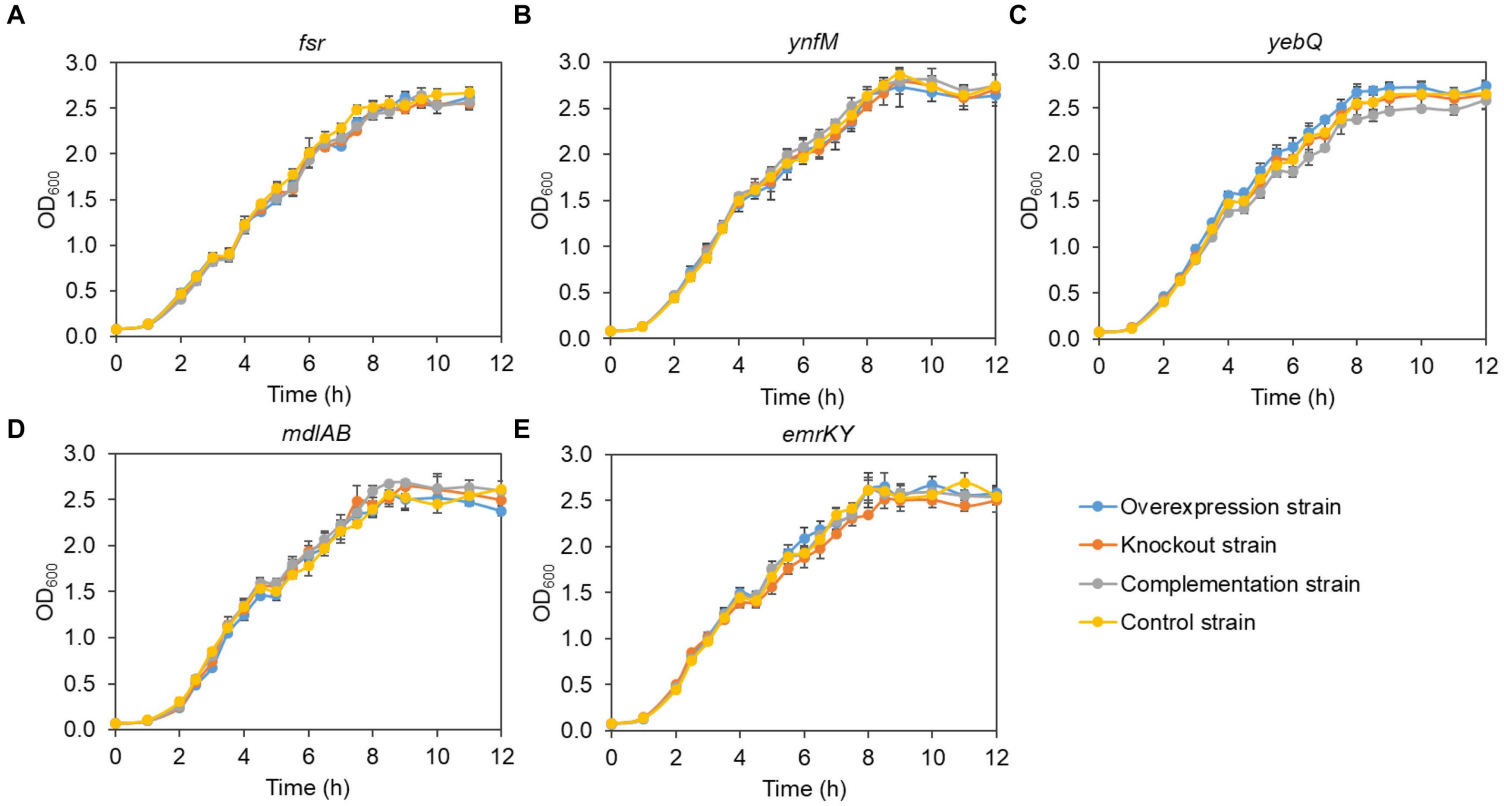
Growth curves of the overexpression strains, knockout strains and complementation strains regarding the transporter genes *fsr* **(A)**, *ynfM* **(B)**, *yebQ* **(C)**, *emrKY* **(D)**, and *mdlAB* **(E)** under the condition without IPTG induction. Data represents mean ± standard deviation of three biological replicates.

**Figure 4:**
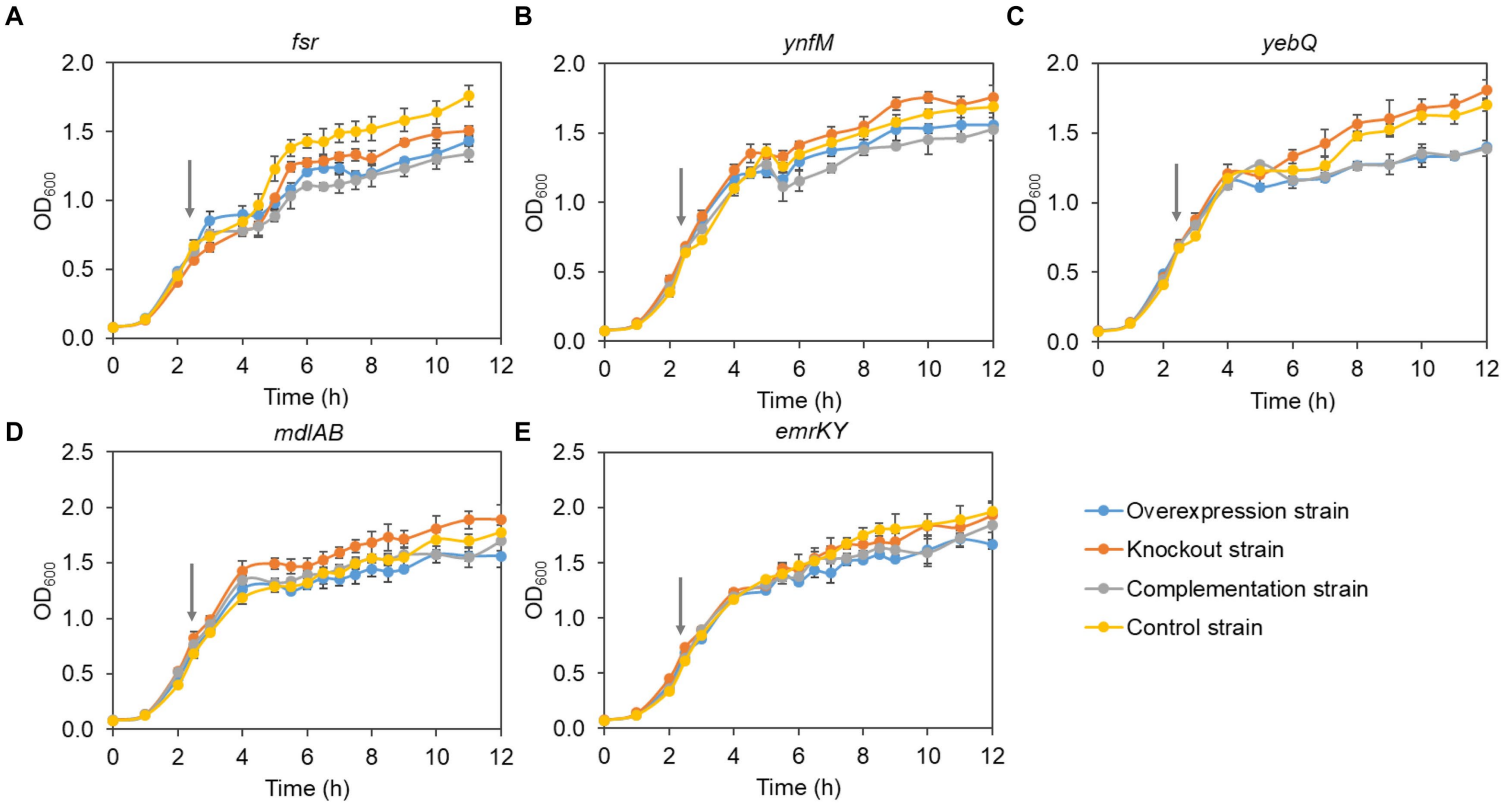
The cell growth of the overexpression strains, knockout strains and complementation strains of transporter genes *fsr* **(A)**, *ynfM* **(B)**, *yebQ* **(C)**, *emrKY* **(D)**, and *mdlAB* **(E)** with the induction of 0.5 mM IPTG. The red arrows indicate the time to add IPTG. Data represents mean ± standard deviation of three biological replicates.

Generally, overexpression of transporters involves extensive folding and translocation of an array of proteins and their translocation to the limited membrane space, thereby causing excessive burden to the central cell metabolism (Jensen et al., 2017; Zhu et al., 2020). To understand how cell growth was affected by the expression levels of the five transporters identified in the present study, we monitored the growth curves of the overexpression, deletion, complementation, and the control strains. To rule out the possible effect of the vector pACYCDuet-1 that was adopted for episomal expression of the transporters, both the deletion and the control strains harbored the empty plasmid. As shown in Figure 3, without IPTG to induce the gene expression, the growth of all the recombinant strains was comparable to that of the control strain, suggesting that single deletion of the transporter genes had no apparent impact on cell growth. In contrast, IPTG induction of the transporter genes and the pathway genes reduced cell growth in the late-log phase and biomass accumulation in the stationary phase for the overexpression and the complementation strains when compared with the deletion and the control strains, although all the strains grew at similar rates at the mid-log phase (Figure 4). In addition, the control strain expressing *ANS* and *3GT* showed greatly reduced biomass compared to the condition without IPTG addition.

### Co-expression of transporter genes was not beneficial for C3G production

**Figure 5:**
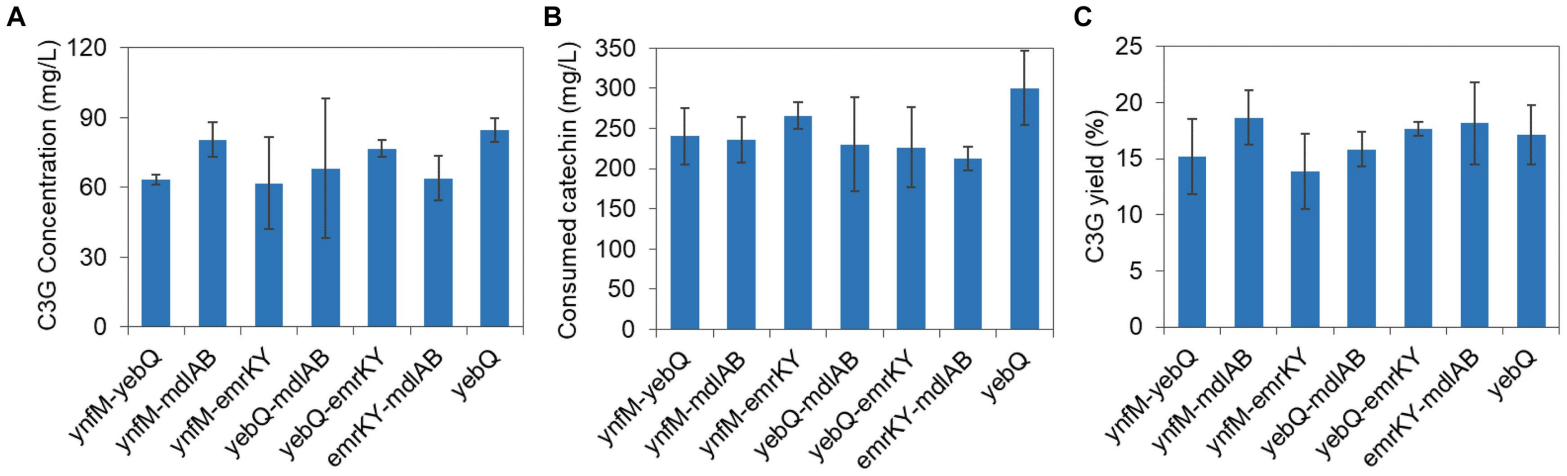
The extracellular C3G production **(A)**, catechin utilization **(B)**, and extracellular C3G yield **(C)** in recombinant strains co-overexpressing two transporter genes among *ynfM*, *yebQ*, *mdlAB*, and *emrKY*. The *yebQ*-overexpressing strain was used as a control. Data represents mean ± standard deviation of three biological replicates.

Given that the overexpression of *ynfM*, *yebQ*, *mdlAB*, and *emrKY* increased C3G production considerably, we explored whether the co-overexpression of these genes could further enhance the bio-production. The *fsr* gene was not included due to the fact that there was not much difference in the yield of extracellular C3G when this gene was overexpressed, deleted, or complemented. Compared with *yebQ* overexpression alone, the co-overexpression of *yebQ* together with *ynfM*, *mdlAB*, or *emrKY* reduced extracellular C3G production (Figure 5A). Similarly, overexpression of *ynfM*, which alone led to the highest extracellular C3G level, showed negative effects when co-expressed with *mdlAB* or *emrKY*. In contrast, the co-overexpression of *mdlAB* and *emrKY* resulted in a level of extracellular C3G production analogous to that when *mdlAB* alone was overexpressed. Co-expression of the transporter genes led to less utilization of catechin but did not cause much variation in the yield of extracellular C3G (Figures 5B,C).

### Double deletion of *mdlAB* and *emrKY* reduced C3G production significantly

**Figure 6:**
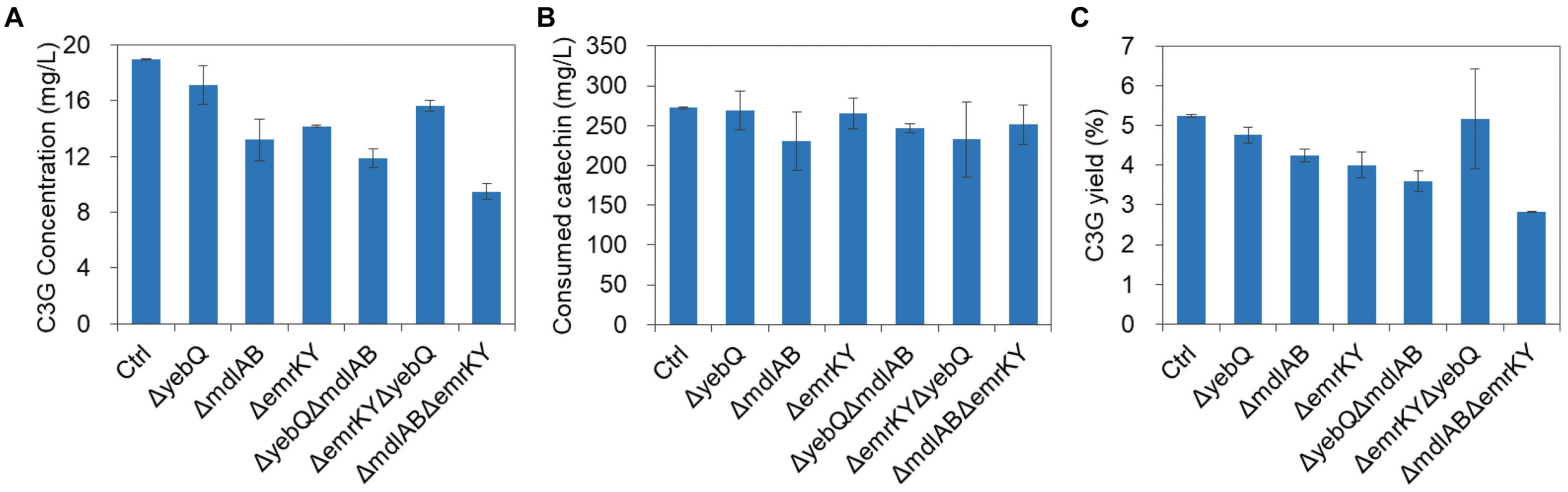
The extracellular C3G production **(A)**, catechin utilization **(B)**, and extracellular C3G yield **(C)** by double knockout strains of *yebQ*, *emrKY*, and *mdlAB*. Data represents mean ± standard deviation of three biological replicates.

Since single deletion of *yebQ*, *mdlAB* and *emrKY* reduced extracellular C3G production titer and the yields, and overexpression and complementation of each individual gene significantly improved both parameters, we wondered how critical these genes are for C3G production. To that end, we constructed double-knockout strains, and found that deletion of *mdlAB*/*emrKY* or *mdlAB*/*yebQ* combination reduced the extracellular C3G concentrations and the yields to levels lower than those in the single-knockout mutants, with the Δ*mdlABΔemrKY* double deletion leading to the largest extent of decline (Figure 6), while the substrate consumption of these two strains was similar to that of the control strain or the corresponding single deletion strains, suggesting that MdlAB and EmrKY are key to the secretion of C3G in *E. coli*. In contrast, Δ*yebQΔemrKY* mutation resulted in less extracellular C3G production than the Δ*yeb* strain but more than the Δ*emrKY* strain. With slightly less catechin utilization, this double deletion strain generated C3G with an extracellular yield comparable to that in the Δ*yebQ* strain (Figure 6).

## Discussion

The bacterial cell envelope is armed with an array of transporters for material exchange, among which the efflux transporters provide protection against adverse environmental factors such as toxins and antimicrobials. Thereby, efflux transporters have been a research focus in the past few decades in the microbial synthesis of valuable compounds aiming at higher tolerance to or better secretion of the products. *E. coli* harbors a panel of MDR transporters, some of which are poorly characterized or even barely annotated. This makes it challenging to understand how anthocyanins, as well as other plant natural products that bear antimicrobial activities, migrate from the cytoplasm to the culture media and how their extracellular microbial synthesis can be improved. In the present study, we analyzed all the known MDR transporters in *E. coli* regarding their competence to enhance C3G efflux, and obtained five transporter genes, i.e., *fsr*, *yebQ*, *ynfM*, *mdlAB*, and *emrKY*, which could prominently strengthen C3G secretion and drive better extracellular synthesis.

Of the five positive transporters, MdlAB belongs to the ABC transporter superfamily and the other four are MFS transporters. Fsr may function as an efflux pump of toxic compounds, as the expression of *fsr* (fosmidomycin resistance) can confer resistance to fosmidomycin, trimethoprim, and carbonyl cyanide m-chlorophenylhydrazone (Fujisaki et al., 1996; Nishino and Yamaguchi, 2001). MdlA is a putative multidrug resistance-like ABC exporter. Its deletion has been reported to increase the total production of free fatty acids but not the extracellular level (Shin and Lee, 2017). MdlB is a putative ABC transporter that confers microbial tolerance to isopentenol (Foo et al., 2014). EmrKY is an efflux pump consisting of an inner membrane subunit and a periplasmic adaptor protein belonging to the major facilitator superfamily (Pasqua et al., 2019). Overexpression of *emrKY* can confer resistance to doxorubicin, rhodamine 6G, benzalkonium, erythromycin, and other toxic chemicals (Nishino and Yamaguchi, 2001). YebQ and YnfM have not been experimentally established as transporters. Overexpression of *yebQ* with its native regulatory gene is reported to confer hypersensitivity to trimethoprim (Nishino and Yamaguchi, 2001). In *E. coli*, YnfM has been predicted to function as an arabinose exporter and its overexpression together with its native regulatory element can sensitize cells to acriflavine (Nishino and Yamaguchi, 2001; Koita and Rao, 2012). The increased sensitivity may be related to the deleterious effect of these genes on cell growth when overexpressed. In accord with this, we observed less biomass accumulation at the stationary phase in cultures overexpressing *yebQ* or *ynfM*. Such damage may cause higher cell permeability, which in turn facilitates catechin uptake and C3G exportation and is unexpectedly beneficial for extracellular C3G production.

Overexpression of *fsr*, *ynfM* or *yebQ* increased C3G production as well as substrate consumption. It is very likely that the timely export of C3G lowers its intracellular concentration, thereby generating a driving force for the continuous uptake of catechin and conversion of catechin to C3G. Indeed, less catechin was utilized when these genes were deleted individually. The strain overexpressing *mdlAB* produced more extracellular C3G with less catechin compared with the control strain. Such a property led to a surge in the yield of extracellular C3G upon the overexpression or complementation of *mdlAB*, suggesting that this gene can be a potential starting point for strain optimization toward better C3G biosynthesis. It is worth mentioning that substrate utilization by *mdlAB*- and *emrKY*-deficient strains was very similar to that by the overexpression strains; nonetheless, the deletion strains produced much lower extracellular C3G than the overexpression strains. Given that catechin and C3G are structurally similar, a possible explanation is that MdlAB and EmrKY are involved in the export of both catechin and C3G, with the former to a lesser extent. With a high level of these transporters, cells on the one hand extrude more catechin to maintain a lower intracellular level for self-protection, and on the other hand export more C3G and correspondingly drives more influx of catechin. The combined effect is better C3G generation.

The co-overexpression of the positive transporter genes did not increase the production titer, suggesting that these genes do not have synergistic or additive roles in C3G biosynthesis. A higher level of efflux transporters can result in the loss of essential metabolites, which is detrimental to the efficiency and stability of the microbial cell factory. Moreover, despite considerable improvement in C3G biosynthesis with the overexpression of *fsr*, *ynfM*, *yebQ*, *mdlAB*, or *emrKY*, deletion of none of these genes completely blocked extracellular C3G accumulation. This clearly demonstrates that the extrusion of C3G is mediated by multiple transporters in a non-specific way, and that there is some extent of redundancy regarding the function of the MDR transporters in C3G efflux. The gene *ynfM* with the highest elevating effect on C3G production upon its overexpression appears not to be the most important transporter gene, as C3G level in its absence was comparable to that in the control strain. In comparison, double deletion of *mdlAB*/*emrKY* or *mdlAB*/*yebQ* further decreased both the extracellular product titer and the yield relative to the single deletion mutation, indicating that MdlAB is more important for C3G biosynthesis.

In summary, the present study identified a panel of MDR transporters closely associated with C3G biosynthesis, i.e., YnfM, YebQ, MdlAB, and EmrKY. These transporters considerably improve both the extracellular C3G concentration and the yield when overexpressed individually, with YnfM demonstrating the best effect. These transporters show redundant roles in C3G export while not displaying any synergistic or additive effect with one another, and it appears that MdlAB and EmrKY in combination are more critical than other transporters to the synthesis of C3G in *E. coli*. As transporters are vital for the intake of essential nutrients and extrusion of toxic metabolites, further production enhancement should focus on the fine regulation of their expression levels via strategies such as variations in the copy numbers and promoter strength, identification of transcription factors, optimization of the sequences in the ribosome binding sites, etc. These approaches will facilitate the establishment of a balance between normal cell growth, appropriate tolerance to the substrate and the product, and an acceptable level of product formation and export. This is the first systematic study investigating the role of all the intrinsic MDR transporters on the microbial production of anthocyanins. Our study potentiates the strategy of transporter identification for the biosynthesis of anthocyanins and related products in *E. coli*. Detailed investigations on the structural and biochemical properties of these transporters will further facilitate the construction of anthocyanin-producing microbes.

## Data availability statement

The original contributions presented in the study are included in the article/Supplementary material, further inquiries can be directed to the corresponding author.

## Author contributions

XW: Writing – original draft, Writing – review & editing, Conceptualization, Formal analysis, Funding acquisition, Project administration. RC: Writing – original draft, Formal analysis, Investigation. PL: Investigation, Writing – original draft, Methodology. JZ: Methodology, Writing – original draft, Conceptualization, Data curation, Funding acquisition, Project administration, Writing – review & editing.
