# Supplementary material for: Identification of Escherichia coli multidrug resistance transporters involved in anthocyanin biosynthesis
Source: Front Microbiol. 2024 Apr 5;15:1357794. doi: 10.3389/fmicb.2024.1357794 (PMC11026601; doi:10.3389/fmicb.2024.1357794)
Supplement: Supplementary file 1 [file Image_1.PDF]

SUPPLEMENTARY MATERIALS

Table S1. Primers used in the present study.

**Primer**	**Sequence (5' to 3' with restriction sites underlined)**	**Purpose**
mdlAB-F	TAAGAAGGAGATATACATATGCGATTATTTGCTCAATTAAGC	Cloning of *mdlAB*
mdlAB-R	GGTTTCTTTACCAGACTCGAGACTATGACAGTGCACAGTGC	
yddA-F	TAAGAAGGAGATATACATATGATAACCATTCCCATTACGC	Cloning of *yddA*
yddA-R	GGTTTCTTTACCAGACTCGAGTTATAAAACCGCGCTAATGTC	
macAB-F	TAAGAAGGAGATATACATATGAAAAAGCGGAAAACCG	Cloning of *macAB*
macAB-R	GGTTTCTTTACCAGACTCGAGTTACTCTCGTGCCAGAGC	
tolC-F	TAATAAGGAGATATACCATGAAGAAATTGCTCCCCA	Cloning of *tolC*
tolC-R	CAGGCGCGCCGAGCTCGAATTCTCAGTTACGGAAAGGGTTATG	
yojIH-F	TAAGAAGGAGATATACATATGGAACTTCTTGTACTTGTCTGG	Cloning of *yojIH*
yojIH-R	GGTTTCTTTACCAGACTCGAGTTACAACGCAATATCCGCC	
yhiHJ-F	TAAGAAGGAGATATACATATGGATAAGAGTAAGCGCC	Cloning of *yhiHJ*
yhiHJ-R	GGTTTCTTTACCAGACTCGAGTTACGCCATTGTCCCAAT	
yajR-F	TAAGAAGGAGATATACATATGAACGATTATAAAATGACGC	Cloning of *yajR*
yajR-R	GGTTTCTTTACCAGACTCGAGTTATGCCTGACGAATTGC	
fsr-F	TAAGAAGGAGATATACATATGGCAATGAGTGAACAACC	Cloning of *fsr*
fsr-R	GGTTTCTTTACCAGACTCGAGTCAGTCTTTATGCCGGTTA	
mdfA-F	TAAGAAGGAGATATACATATGCAAAATAAATTAGCTTCCG	Cloning of *mdfA*
mdfA-R	GGTTTCTTTACCAGACTCGAGTTACCCTTCGTGAGAATTTCC	
mdtG-F	TAAGAAGGAGATATACATATGTCACCCTGTGAAAATG	Cloning of *mdtG*
mdtG-R	GGTTTCTTTACCAGACTCGAGTCAGTTCGATACCTGGGG	
ydeA-F	TAAGAAGGAGATATACATATGACAACAAACACTGTTTCC	Cloning of *ydeA*
ydeA-R	GGTTTCTTTACCAGACTCGAGCTATTGCGTCTGTTCTTCG	
ydeE-F	TAAGAAGGAGATATACATATGAACTTATCCCTACGACG	Cloning of *ydeE*
ydeE-R	GGTTTCTTTACCAGACTCGAGTCAACAAAGCGCGGG	
ynfM-F	TAAGAAGGAGATATACATATGAGCCGTACTACAACTG	Cloning of *ynfM*
ynfM-R	GGTTTCTTTACCAGACTCGAGTCAGGCGTGCAGACG	
ydhC-F	TAAGAAGGAGATATACATATGCAACCTGGGAAAAGA	Cloning of *ydhC*
ydhC-R	GGTTTCTTTACCAGACTCGAGTCAGTGTGATTCGCTATGAG	
ydiM-F	TAAGAAGGAGATATACATATGAAAAATCCCTATTTCCCTACC	Cloning of *ydiM*
ydiM-R	GGTTTCTTTACCAGACTCGAGTTACCCACCCGGAGC	
yebQ-F	TAAGAAGGAGATATACATATGCCAAAAGTTCAGGC	Cloning of *yebQ*
yebQ-R	GGTTTCTTTACCAGACTCGAGTTATGCCCTGGATCGTG	
bcr-F	TAAGAAGGAGATATACATATGACCACCCGACAGC	Cloning of *bcr*
bcr-R	GGTTTCTTTACCAGACTCGAGTCACCGTTTTTTCGGCC	
emrKY-F	TAAGAAGGAGATATACATATGGAACAGATTAATTCAAATAAAAA	Cloning of *emrKY*
emrKY-R	GGTTTCTTTACCAGACTCGAGTCACCCAACGCCTTTC	
emrAB-F	TAAGAAGGAGATATACATATGAGCGCAAATGCGG	Cloning of *emrAB*
emrAB-R	GGTTTCTTTACCAGACTCGAGTTAGTGCGCACCGC	
emrD-F	TAAGAAGGAGATATACATATGAAAAGGCAAAGAAACG	Cloning of *emrD*
emrD-R	GGTTTCTTTACCAGACTCGAGTTAAACGGGCTGCCCC	
mdtL-F	TAAGAAGGAGATATACATATGTCCCGCTTTTTGATTTG	Cloning of *mdtL*
mdtL-R	GGTTTCTTTACCAGACTCGAGTCAAGCGTGGTGATGG	

hsrA-F	TAAGAAGGAGATATACATATGAGCGATAAAAAGAAGCG	Cloning of *hsrA*
hsrA-R	GGTTTCTTTACCAGACTCGAGTTACTCCGATTCTGATGGAAC	
mdtA-F	TAAGAAGGAGATATACATATGAAAGGCAGTTATAAATCCCG	Cloning of *mdtABCD*
mdtABCD-R1	TTACGCCTCCTCTTCATG	
mdtABCD-F2	GCCCGTCATGAAGAGG	
mdtABCD-R2	GGTTTCTTTACCAGACTCGAGTCATTGCGCGCTCC	
mdtM-F	TAAGAAGGAGATATACATATGCCACGTTTTTTTACCC	Cloning of *mdtM*
mdtM-R	GGTTTCTTTACCAGACTCGAGTCACTGCTCCTCCACTAG	
mdtK-F	TAAGAAGGAGATATACATATGCAGAAGTATATCAGTGAAGCG	Cloning of *mdtK*
mdtK-R	GGTTTCTTTACCAGACTCGAGTTAGCGGGACGCTCG	
acrAB-F	TAAGAAGGAGATATACATATGAACAAAAACAGAGGGT	Cloning of *acrAB*
acrAB-R	GGTTTCTTTACCAGACTCGAGTCAATGATGATCGACAGTATG	
cusCFBA-F	TAAGAAGGAGATATACATATGTCTCCTTGTAAACTTCTGCC	Cloning of *cusCFBA*
cusCFBA-R	GGTTTCTTTACCAGACTCGAGTTATTTCCGTACCCGATGTCGG	
acrD-F	TAAGAAGGAGATATACATATGGCGAATTTCTTTATTGATCG	Cloning of *acrD*
acrD-R	GGTTTCTTTACCAGACTCGAGTTATTCCGGGCGCGG	
acrEF-F	TAAGAAGGAGATATACATATGACGAAACATGCCAGG	Cloning of *acrEF*
acrEF-R	GGTTTCTTTACCAGACTCGAGTTATCCTTTAAAGCAACGGCG	
emrE-F	TAAGAAGGAGATATACATATGAACCCTTATATTTATCTTGGTG	Cloning of *emrE*
emrE-R	GGTTTCTTTACCAGACTCGAGTTAATGTGGTGTGCTTCGT	
sugE-F	TAAGAAGGAGATATACATATGATGAAACGCCTTATCGTTC	Cloning of *sugE*
sugE-R	GGTTTCTTTACCAGACTCGAGTTATTGAGTGCTGAGTTTCAGACC	
mdtJI-F	TAAGAAGGAGATATACATATGTATATTTATTGGATTTTATTAGGTC	Cloning of *mdtJI*
mdtJI-R	GGTTTCTTTACCAGACTCGAGTCAGGCAAGTTTCACCATGAT	
mdtNOP-F	TAAGAAGGAGATATACATATGGAAAGTACGCCGAAAAA	Cloning of *mdtNOP*
mdtNOP-R	GGTTTCTTTACCAGACTCGAGTTATTTTTTCTCGACGACGGG	
pTarget-mdlAB-F-speI	TAATACTAGTCGCCGTGGCGTAAACCGCTGGTTTTAGAGCTAGAAATAGCAAGTTAAA	Cloning of sgRNA of *mdlAB*
ynfM-BamHI-F	CATCACCACAGCCAGGATCCGATGAGCCGTACTACAACTGTTG	Co-overexpression of *ynfM* with *yebQ*, *mdlAB*, or *emrKY*
ynfM-NotI-R	CTTAAGCATTATGCGGCCGCTCAGGCGTGCAGACGACGATG	
yebQ-BamHI-F	CATCACCACAGCCAGGATCCGATGCCAAAAGTTCAGGCCGAC	Co-overexpression of *yebQ* with *mdlAB* or *emrKY*
yebQ-NotI-R	CTTAAGCATTATGCGGCCGCTTATGCCCTGGATCGTGGCTG	
emrKY-BamHI-F	CATCACCACAGCCAGGATCCGATGGAACAGATTAATTCAAATAAAAAAC	Co-overexpression of *emrKY* with *mdlAB*
emrKY-NotI-R	CTTAAGCATTATGCGGCCGCTCACCCAACGCCTTTCGC	
pTarget-R-EcoRI	CTCTAGAGAATTCAAAAAAAGCACCGACT	Reverser primer to construct sgRNA for all the deletion
mdlAB-F1-EcoRI	TTTGGGAATTCGGTGGCGCGTATTGATGCTC	Construction of homology arm of *mdlAB*
mdlAB-R1	CGGCCCCTGGCTTCCGTCAGTGCCG	
mdlAB-F2	GGAAGCCAGGGGCCGCTGCTTATCAGC	
mdlAB-R2-XhoI	GGGCCCTCGAGTGCCACTCTGTAACGGGCGATCA	
pTarget-fsr-F-speI	TAATACTAGTATTGGCAACGACCATGGCATGTTTTAGAGCTAGAAATAGCAAGTTAAA	Construction of sgRNA of *fsr*
fsr-F1-EcoRI	TTTGGGAATTCGGCGCAACGCCGAGGATAGAGCCC	Construction of homology arm of *fsr*
fsr-R1	CCGCTGCCCGTATGGCTTCCGGCGGG	

fsr-F2	GCCATACGGGCAGCGGATAAATCGCCAGAATC	
fsr-R2-XhoI	GGGCCCTCGAGCGCCAAAAATGCCCATGATG	
pTarget-ynfM-F-speI	TAATACTAGTGGCCTTTAGCGCGTTTTGCGGTTTTAGAGCTAGAAATAGCAAGTTAAA	Construction of sgRNA of *ynfM*
ynfM-F1-EcoRI	TTTGGGAATTCGCCTTGATTGGGCTTTCG	Construction of homology arm of *ynfM*
ynfM-R1	CCCCAGCGCAGCATAACCCCCGTCGA	
ynfM-F2	GGTTATGCTGCGCTGGGGGGTGTTTTC	
ynfM-R2-XhoI	GGGCCCTCGAGCGGCAACAACCAGCGC	
pTarget-yebQ-F-speI	TAATACTAGTGCGCTGGGCAGGTCGAAACGGTTTTAGAGCTAGAAATAGCAAGTTAAA	Construction of sgRNA of *yebQ*
yebQ-F1-EcoRI	TTTGGGAATTCAGTGCTAGAATCATACCCCTGTTG	Construction of homology arm of *yebQ*
yebQ-R1	GCGAAACCACTGCAGCAATTGTCGGCCC	
yebQ-F2	TTGCTGCAGTGGTTTCGCTCAGGGG	
yebQ-R2-XhoI	GGGCCCTCGAGCCGCTACTCTGACCCAGTAG	
pTarget-emrKY-F-speI	TAATACTAGTACTCCGGCACCATTAACCGGGTTTTAGAGCTAGAAATAGCAAGTTAAA	Construction of sgRNA of *emrKY*
emrKY-F1-EcoRI	TTTGGGAATTCGGCATAAATGTCACAGAACGCC	Construction of homology arm of *emrKY*
emrKY-R1	CCTCCATGCCATCACCTCGTTTGGTGTAGCA	
emrKY-F2	CGAGGTGATGGCATGGAGGTCACGGTTG	
emrKY-R2-SalI	GGGCCGTCGACAGGTGCCTATGCCTATTGG	

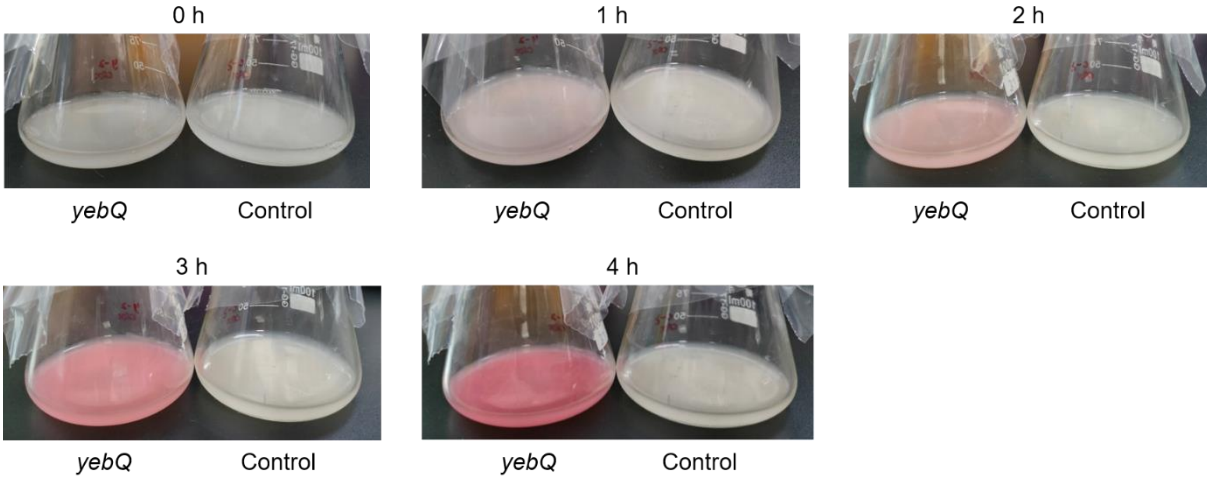

Figure S1. The appearance of the fermentation products by *yebQ*-overexpressing strain and the control strain at different time points during the fermentation process.
